# Exercise-Induced Changes in Bioactive Lipids Might Serve as Potential Predictors of Post-Exercise Hypotension. A Pilot Study in Healthy Volunteers

**DOI:** 10.3390/cells9092111

**Published:** 2020-09-16

**Authors:** Miriam C. Wolters, Julia Schmetzer, Christine V. Möser, Lisa Hahnefeld, Carlo Angioni, Dominique Thomas, Nerea Ferreirós, Gerd Geisslinger, Ellen Niederberger

**Affiliations:** 1 Pharmazentrum Frankfurt/ZAFES, Institut für Klinische Pharmakologie, Klinikum der Goethe-Universität Frankfurt, Theodor Stern Kai 7, 60590 Frankfurt am Main, Germany; miriam_wolters@gmx.de (M.C.W.); juliasch@yahoo.de (J.S.); chmoeser@hotmail.com (C.V.M.); hahnefeld@med.uni-frankfurt.de (L.H.); angioni@em.uni-frankfurt.de (C.A.); Thomas@med.uni-frankfurt.de (D.T.); ferreirosbouzas@em.uni-frankfurt.de (N.F.); geisslinger@em.uni-frankfurt.de (G.G.); 2 Fraunhofer Institute for Molecular Biology and Applied Ecology (IME), Branch for Translational Medicine and Pharmacology TMP, Theodor Stern-Kai 7, 60590 Frankfurt am Main, Germany

**Keywords:** bioactive lipids, exercise, blood pressure, post-exercise hypotension

## Abstract

Post-exercise hypotension (PEH) is the phenomenon of lowered blood pressure after a single bout of exercise. Only a fraction of people develops PEH but its occurrence correlates well with long-term effects of sports on blood pressure. Therefore, PEH has been suggested as a suitable predictor for the effectivity of exercise as therapy in hypertension. Local vascular bioactive lipids might play a potential role in this context. We performed a cross-over clinical pilot study with 18 healthy volunteers to investigate the occurrence of PEH after a single short-term endurance exercise. Furthermore, we investigated the plasma lipid profile with focus on arachidonic acid (AA)-derived metabolites as potential biomarkers of PEH. A single bout of ergometer cycling induced a significant PEH in healthy volunteers with the expected high inter-individual variability. Targeted lipid spectrum analysis revealed significant upregulation of several lipids in the direct post-exercise phase. Among these changes, only 15- hydroxyeicosatetranoic acid (HETE) correlated significantly with the extent of PEH but in an AA-independent manner, suggesting that 15-HETE might act as specific PEH-marker. Our data indicate that specific lipid modulation might facilitate the identification of patients who will benefit from exercise activity in hypertension therapy. However, larger trials including hypertonic patients are necessary to verify the clinical value of this hypothesis.

## 1. Introduction

According to the World Health Organization (WHO), hypertension has a very high prevalence world-wide and causes about 7.5 million premature deaths per year [1]. Lowering blood pressure by lifestyle changes and/or pharmacological treatment reduces the occurrence of severe or fatal cardiovascular events [2,3]. Several reports also indicate that regular exercise by hypertonic patients beneficially influences blood pressure [4,5] with lower costs and avoiding the side effects that occur with drug treatment [6]. Unfortunately, only 75% of the hypertonics respond to exercise therapy [7]. Thus, identification of individuals who are susceptible to modification by exercise would enable a rapid start of therapy with an adequate treatment regimen. Post-exercise hypotension (PEH) is the phenomenon of lowered systolic blood pressure after a single bout of exercise, which is suggested as a suitable predictor of the potential success of long-term exercise therapy in hypertonia [8,9,10]. Thus far, the molecular mechanisms of PEH are not fully understood. It has been postulated that the concentration of local vasodilators contributes to lowering of blood pressure [11]. However, the distinct composition of these mediators is still unclear and compounds suitable as biomarkers for PEH have not been identified. Besides their prominent role in inflammatory processes, eicosanoids derived from arachidonic acid metabolism might play an important role in the regulation of blood pressure [12]. Epoxyeicosatrienoic acid (EET), dihydroxyeicosatrienoic acid (DHET) and 20-hydroxyeicosatetranoic acid (HETE) are AA-derived products of the CYP pathway. While EETs and DHETs have mostly been associated with antihypertensive effects, 20-HETE is reported to have vasoconstrictive and hypertensive actions (reviewed in [12,13,14]). Lipoxygenase-derived HETEs also show vasoactive properties and have been associated with both vasodilation and vasoconstriction. ThreeLOX isoforms generating 5-, 12- and 15-HETE are expressed in vascular endothelial cells. 5-LOX contributes to the regulation of inflammatory processes through synthesis of leukotrienes. Additionally, 5-LOX-derived 5-HETE has been linked with vasoconstrictive properties [15,16,17,18,19]. On the other hand, 15-LOX products exert either vasodilatory or vasoconstricting effects depending on the concentration, vascular type and other local factors [20,21]. Metabolites generated by COX activity, such as PGE2 and TXA2, are well known for their role in inflammatory processes and the cardiovascular system (reviewed in [22]).

Thus far, there have been only few initial studies that investigated arachidonic acid metabolites in plasma, serum or muscle tissue after different types of exercise. The results of these studies showed exercise-induced regulation of various AA metabolites [23,24,25,26,27]. However, none of these reports assessed the function of these mediators in correlation with induction of PEH. Only one paper discussed CYP450 products as potential vasodilating agents after endurance exercise [23] and some data hint towards induction of vasodilating prostaglandins by muscle contraction [28], but their contribution to PEH is questionable, since COX inhibitors were not able to prevent PEH [29].

The aim of this study was to elucidate changes in bioactive lipids after a single bout of endurance exercise, as well as the potential impact of lipid modification on the development of PEH. Since COX-derived and CYP450-derived AA products have already been considered as potential exercise-induced vasodilators, we focused in particular on lipoxygenase-derived HETEs. We hypothesized that exercise-induced changes in HETE levels might correlate with the induction of PEH.

## 2. Materials and Methods

### 2.1. Study Design

**Figure 1:**
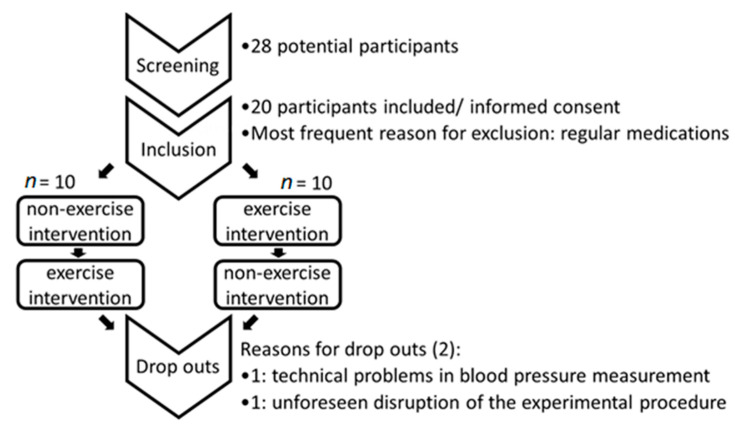
Flow chart of participant choice.

From 28 applicants, we included 20 healthy volunteers, (for demographic data see Table 1, Figure 1) who fit the inclusion and exclusion criteria, for this prospective crossover trial. All subjects were properly informed of the study procedures and gave signed written consent. The study was approved by the local ethical committee of Goethe University Frankfurt (497/14, approved 2015). The study was performed in accordance with the ethical standards as laid down in the 1964 Declaration of Helsinki and its later amendments or comparable ethical standards. During the study, we had two drop-outs (both male): one because of technical problems with blood pressure measurement and one because of disruption of the experimental procedure. Therefore, data analysis was performed for 18 participants. All participants were invited to two intervention days, one with exercise and one without exercise. The series of experimental procedures was randomly chosen: 50% of the volunteers started with the exercise intervention and the other 50% with the non-exercise control intervention.

### 2.2. Endpoints of the Study

Endpoints of the study were the determination of a panel of bioactive lipids after a single bout of exercise as well as correlation of plasma lipid changes and the magnitude of PEH.

### 2.3. Inclusion Criteria

- Age between 18 and 35 years.
- Caucasian.
- Written consent.
- Sufficient body fitness to perform exercise intervention.

### 2.4. Exclusion Criteria

- Existing diseases that are associated with a health risk in combination with exercise at 80% of maximal heart rate (e.g., Angina pectoris, malign arrhythmia; bronchial asthma).
- Existing diseases which hinder exercise performance (e.g., peripheral arterial occlusion, severe respiratory disease, painful disease of musculoskeletal system).
- Existing disease with increased risk of injury during exercise (e.g., epilepsy; disturbance of equilibrium).
- Pathological changes in ECG.
- Pathological blood count, increased inflammatory parameters in screening examination.
- Pregnancy.
- Acute disease in the last 14 days before exercise intervention.
- Psychiatric disorder which does not allow voluntary oral consent.
- Chronic drug administration (except oral contraceptives) or drug administration in the last 48 h prior to exercise intervention.
- Known hypertonia or hypertonia as diagnosed in screening examination.
- Consumption of caffeine in the last 12 h before intervention.
- Intake of licorice or grapefruit 48 h before intervention.

### 2.5. Exercise Protocol, Blood Pressure Determination and Blood Sample Preparation

All participants started with the study protocol at the same time between 8 and 9 a.m., to exclude the influence of circadian rhythms. On one of the two intervention days, they carried out an exercise protocol (“training intervention”); on the other study day, they went through a sedentary protocol (“control intervention”). Half of the participants started with the control day, the other half with the intervention day to prevent sequence specific effects. They were advised to have the same light breakfast and sufficient liquid uptake 60–120 min prior to both interventions. Furthermore, the volunteers should not have executed any sports or heavy work up to 12 h before intervention.

Exercise intensity was performed at 80% of the theoretical age-adapted heart rate, which was calculated with the formula: Age-adapted heart rate = (220 − age) × 0.8.

At the beginning of the intervention, a venous catheter was inserted that allowed repeated blood withdrawal. Blood pressure was determined at time point −60 min before the exercise intervention started. The −60 min time point was, therefore, defined as a baseline for all measurements. On the intervention day, the following measurements were performed every 5 min for 30 min before exercise, directly after exercise and then every 5 min until the end of the experiment at 180 min. On the control intervention day, blood pressure was measured every 5 min until the end of the experiment. Because of natural variations in blood pressure measurement, calculations were performed using the average of values within a 15 min period (e.g., the value at time point −60 min constitutes the mean of values at −60, −55 and −50 min before start of exercise).

Endurance exercise on an ergometer (Proxomed Kardiomed Diagnostic Cycle, proxomed Medizintechnik, Alzenau, Germany) started at −30 min for 30 min. On the day of non-exercise control intervention, the participants remained sedentary during the whole procedure.

During the training intervention, blood samples were taken before exercise (−60 min) and then at 0; 5; 10; 30; 60; 120 and 180 min and directly prepared for analysis. During the control intervention, blood samples were drawn at −60, 0, 60, 120 and 180 min (Supplementary Figure S1).

### 2.6. Blood Withdrawal and Plasma Preparation

Blood withdrawal was performed with an indwelling venous catheter in the Vena cubitalis. Maximally 12 mL of blood was taken at each time point. The catheter was removed after maximally 6 h. A break of at least 14 days was planned between the first and the second intervention to avoid problems with the injection site or high blood loss. Blood was collected in an EDTA-tube, which was inverted several times. A small amount of blood (500 µL) was separated for cell counting before centrifugation at 2.000× *g* at room temperature for 10 min. The plasma supernatant was withdrawn and immediately stored in aliquots at −80 °C until further analysis.

### 2.7. Measurement of Hematocrit

Hematocrit was determined using a pocH-100i (Sysmex, Norderstedt, Germany) according to manufacturer’s instructions.

### 2.8. Sample Preparation and LC-MS/MS Analysis of Lipid Mediators

Lipid mediators were analyzed using liquid chromatography tandem-mass spectroscopy (LC-MS/MS). All standards and several of their deuterated derivatives were obtained from Cayman Chemicals (Ann Arbor, MI, USA): hydroxyeicosatetraenoic acids (5(S)-HETE-d8, 5(S)-HETE, 12(S)-HETE, 15(S)-HETE and 20(S)-HETE), arachidonic acid, arachidonic acid-d8, epoxyeicosatetraenoic and dihydroxyeicosatrienoic acids (5,6-EET; 8,9-EET; 11,12-EET; 14,15-EET; 5,6-EET-d11; 8,9-EET-d11; 11,12-EET-d11; 14,15-EET-d11; 5,6-DHET; 8,9-DHET; 11,12-DHET;14,15-DHET; 8,9-DHET-d11; 11,12-DHET-d11 and 14,15-DHET-d11), prostaglandin E2 (PGE2) and prostaglandin E2-d4 (PGE2-d4), and thromboxane B2 (TXB2) and TXB2-d4. Acetonitrile, water, methanol (LC-MS grade) and butylated hydroxytoluene (BHT) were purchased from Carl Roth (Karlsruhe, Germany). Formic acid (pro analysis) was purchased from VWR (Darmstadt, Germany). Acetic acid was obtained from fisher scientific (Schwerte, Germany) and ammonia from Merck (Darmstadt, Germany).

The LC-MS/MS system consisted of a 5500 QTrap mass spectrometer (Sciex, Darmstadt, Germany), operating in negative ESI mode, an Agilent 1200 HPLC system (Agilent, Waldbronn, Germany) and an HTC Pal autosampler (Chromtech, Idstein, Germany).

Sample extraction of HETE, EET and DHET was performed using liquid-liquid extraction: 200 µL of plasma were gently mixed with 20 µL of methanol and 20 µL of internal standard solution and extracted twice with 600 µL ethyl acetate. Samples for standard curve and quality control were prepared similarly: 200 µL PBS, 20 µL of standard solution and 20 µL internal standard solution were mixed and extracted with ethyl acetate.

The organic phase was removed at 45 °C under a gentle stream of nitrogen. The residues were reconstituted in 50 µL of methanol:water:BHT (50:50:10^−4^, *v/v/v*) prior to injection into the LC-MS/MS system. Chromatographic separation was achieved using a Gemini NX C18 column (150 mm × 2 mm ID, 5 µm, Phenomenex, Aschaffenburg, Germany) with a precolumn of the same material. A linear gradient was employed at a flow rate of 0.5 mL/min and a total run time of 17.5 min. Mobile phases were A water:ammonia (100:0.05, *v/v*) and B acetonitrile:ammonia (100:0.05, *v/v*). The gradient started from 85% A, changed to 10% A within 12 min, was maintained for 1 min, and shifted back to 85% A 0.5 min following 3.5 min equilibration.

Arachidonic acid was extracted as described above from 100 µL plasma. The residues were reconstituted in 100 µL of methanol:water:BHT (50:50:10^−4^, *v/v/v*) before analysis. Chromatographic separation of arachidonic acid was achieved on a MercuryMS Synergi Hydro-RP column (20 × 2 mm ID, 2.5 µm, Phenomenex, Aschaffenburg, Germany) with mobile phase A and B water and acetonitrile with 0.0025% acetic acid, respectively. The linear gradient started at 60% A, changed to 0% A in 1.5 min, was maintained for 1 min, and shifted back to 60% A 1 min following 2.5 min of equilibration.

Prostanoids (PGE2, thromboxane B2) were analysed from 50 µL of plasma as described previously [30] using an Eksigent NanoLC 2D Ultra System (Eksigent, part of AB Sciex, Redwood City, CA, USA) coupled to a 5500 QTrap mass spectrometer (AB Sciex, Darmstadt, Germany), operating in negative ion mode.

All data were acquired using Analyst software v. 1.6.2 and quantitation was performed by MultiQuant software v. 3.0 (both Sciex, Darmstadt, Germany) using the internal standard method (isotope-dilution mass spectrometry). Calibration curves were calculated by linear regression with 1/x or 1/x^2^ weighting and acceptance criteria were applied as described previously [31].

### 2.9. Statistics

The study was planned as a pilot study to gain an insight into potential lipid regulations during and after exercise and their impact on development of PEH. The planning of the number of cases and of the statistical analysis was carried out after expert consultation at the Institute for Biometry and Biostatistics at the Goethe University Frankfurt. As this is a pilot study, the following estimates were only theoretical approximations. We also made normal distribution assumptions for the planning of case numbers: with a mean difference of the percentage differences from the initial value (Δtx − Δt0) = 1, it was assumed that the standard deviation sigma is at most 1.5 times this value. These assumptions resulted in a required number of 20 subjects if a power 1-ß of at least 0.8 is to be achieved with the one-sample t-test at a significance level of α = 0.05.

Statistical analysis was performed using SPSS 27.0. for all calculations and graphs, except Friedman test with post hoc tests, which was done by “BIAS for windows v. 11.1”. α < 0.05 was defined as significant in all statistical procedures. Data were checked for normality with Kolmogorov-Smirnov Test. If there were doubts concerning normality, Shapiro-Test and visual inspection of histogram data were performed additionally.

The design of the study included two factors (exercise vs non-exercise) and multiple time-points for each factor. Because of high inter-individual differences in lipid values as well as in blood pressure, values of the time courses were normalized to their corresponding baseline value. For measurements with normal distributed data, we applied a repeated measures ANOVA. A Mauchly test was performed to assess sphericity. If sphericity could not be assumed, Greenhouse-Geisser correction was done. For multivariate testing, Wilk’s lambda was calculated.

Potential differences between exercise and control intervention were analyzed using pairwise comparison with Bonferroni correction for the factor “intervention” at the time points 0, 30, 60 and 120 min.

In case of major deviations from normal distribution, which occurred with plasma lipid levels of AA and its metabolites, there is no established statistical non-parametric analogue for between groups comparison of multiple time points in a cross-over design. Therefore, we only compared lipid values of exercise versus sedentary control intervention at a specific reference point (RP) using the Wilcoxon signed rank test for dependent samples. As RP, we determined the time point at which changes in the hematocrit as indicator for exercise-induced fluid shift were diminished. The null hypothesis was that the median in differences between exercise and sedentary control intervention was 0. An exact calculation of significance was performed and *p* < 0.05 was chosen as significance level for a two-tailed test. The z-value was calculated and Pearson’s r was calculated by r = |z/$\sqrt{n}$| to evaluate the effect size [32] of differences between the treatments.

To use the additional information of the multiple time points in this pilot study, the Friedman test with Conovert-Bonferroni-Holm post-hoc test was performed to compare lipid values after exercise and sedentary control intervention to their corresponding baseline values.

In order to compare the amount of post-exercise reduction of blood pressure between the participants and to relate it to plasma lipid levels, the blood pressure 30 min after cessation of exercise (mean of measurements at 30, 35 and 40 min after exercise) was defined as parameter for PEH. This time point has been repeatedly reported for the strongest blood pressure regulations after exercise [33,34,35] and showed significant decreases of systolic blood pressure in our study.

Pearson’s product moment correlation was done to empirically test for possible relations between PEH and the extent of lipid liberation after exercise. Pearson’s r was used to evaluate the size of correlation. As Pearson’s correlation is sensitive to extreme values, manual inspection of the correlation plot was done to exclude dependence on extreme values.

Since small sample sizes and deviations from normal distribution may bias correlations, a bootstrapping method was conducted to further validate the significant Pearson correlations [36,37] and the bias corrected accelerated 95% confidence interval (95% BCa) was determined [36,38]. A positive or negative correlation can be assumed if upper and lower boundaries of the confidence interval do not intersect with zero. The number of samples was set as “2000.”

To assess the unique effect of 15-HETE on PEH, bivariate semi-partial correlations with AA, 5-HETE, 12-HETE, 20-HETE and BMI were performed to evaluate them as confounding factors. Because of a homogenous age group and the small sample size, age and gender as separate factors were not considered.

## 3. Results

### 3.1. Effects of Cycling Exercise on Heart Rate and Blood Pressure

**Figure 2:**
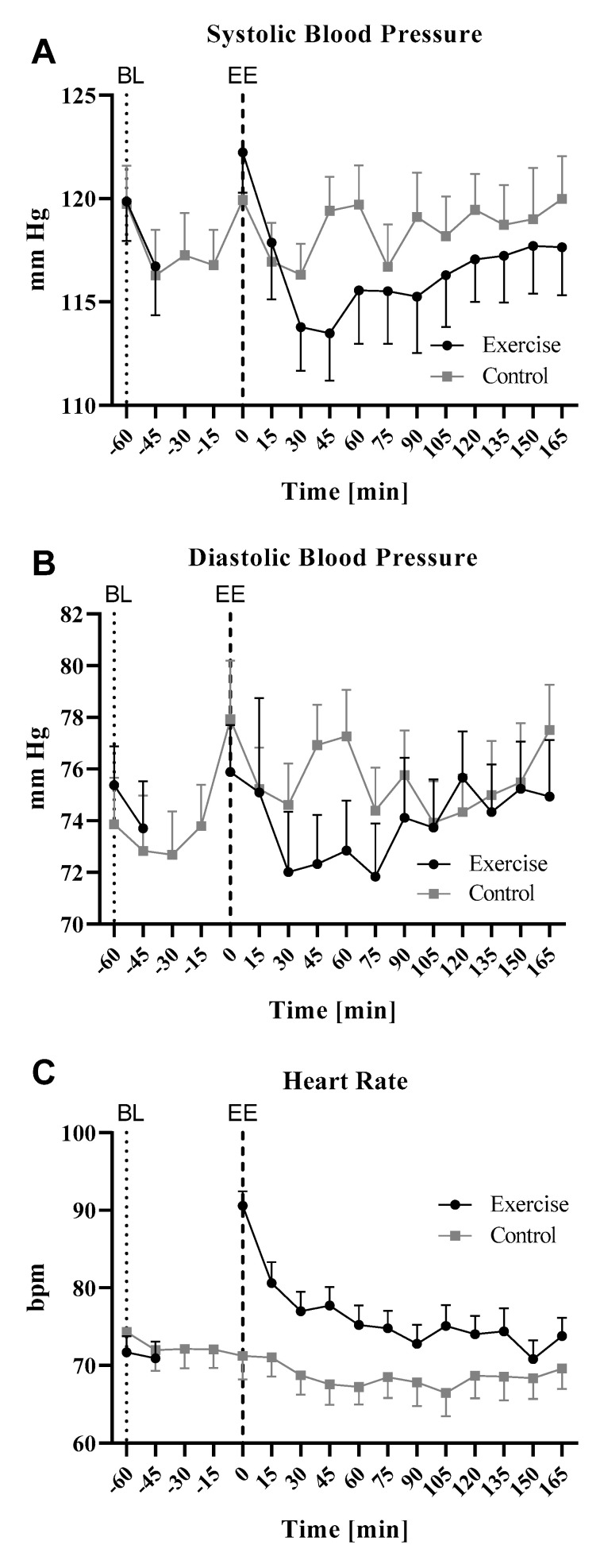
Time course of blood pressure and heart rate during exercise and control intervention, (**A**): Mean systolic blood pressure [mm Hg], (**B**): Mean diastolic blood pressure [mm Hg], (**C**): Heart rate [beats per minute, bpm]. Baseline values (BL) were determined at −60 min at both study days. Since it was not possible to perform measurements during ergometer cycling, there is a gap in the exercise intervention graph during this period, black: exercise intervention grey: sedentary control intervention; values are expressed as means ± SE, *n* = 18, EE: end of exercise. Corresponding values are indicated in Supplementary Table S1.

Heart rate and blood pressure were determined during the control and the training interventions at the indicated time points (Figure 2, Supplementary Table S1). Baseline values were almost identical on both intervention days.

Systolic blood pressure (SBP) is usually used to determine PEH. SBP was slightly elevated directly after exercise but dropped during the direct and early post-exercise time period to a nadir mean of about 6% lower at 30–45 min after exercise cessation, compared to baseline. From 45 min after exercise cessation, SBP slowly rose again up to approximately baseline levels at the end of the study session. During the control intervention, systolic blood pressure remained relatively stable with slight undulating changes (Figure 2A). For statistical analysis, we calculated relative values for systolic blood pressure and set the baselines to 1 (Table 2). Repeated measures ANOVA with pairwise comparison for both treatment interventions revealed a significant difference between exercise and control intervention at +60 min after exercise (Mauchly-w: 0.755, *p* = 0.428; Inner-subject effects (time * intervention; sphericity assumed): F = 3.405; *p* = 0.01; Inner-subject contrasts: (time * intervention): +60 min vs baseline: F = 4.524 *p* = 0.041). Repeated measures ANOVA with comparison to baseline for both interventions separately revealed a significant decline in blood pressure compared to baseline in the exercise intervention at +30 min after cessation of exercise (Mauchly-W: 0.394; *p* = 0.112; Wilks-Lambda: F = 9.297; *p* = 0.001; Inner-subject effects (sphericity assumed): F = 10.849; *p* < 0.001; Pairwise comparison (time points versus baseline): significant at +30 min with *p* < 0.0001). In the sedentary control intervention group, there were no significant changes in the pairwise comparison to baseline (Mauchly-W: 0.752; *p* = 0.885; Wilks-Lambda: F = 1.853; *p* = 0.175). In accordance with these results and former reports, the systolic blood pressure reduction at time point +30 min was defined as the extent of PEH in our study. The diastolic blood pressure (DBP) changes after exercise were more variable than the systolic. However, there was a tendency towards lower DBP after exercise with a nadir at about 30–45 min after cessation of exercise (~3–5%) and an increase towards baseline values afterwards. During the control intervention, DBP slightly but constantly increased until the end of the observation period (Figure 2B). As expected, the heart rate increased during exercise and was 30% higher compared to baseline directly after exercise. In the follow-up period, this value decreased to ~ +10% within the first 15 min post-exercise and remained within this range until the end of the experiment. A slight decrease in heart rate occurred in the non-exercise control intervention throughout the observation time. Overall, the heart rate was higher during the exercise intervention than during the control intervention (Figure 2C).

As described, we defined the systolic blood pressure at the time point +30 min after cessation of exercise as the quantification parameter for PEH. According to this definition, we observed a significant mean PEH after ergometer cycling with a mean systolic blood pressure reduction of 6.1 mmHg (Wilk’s Lambda *p* = 0.001; Greenhouse-Geisser test for within-subject effects: *p* < 0.001; Pairwise comparisons with Bonferroni corrections *p* < 0.001). However, the extent of PEH differed strongly between the single participants with reductions in blood pressure varying between −0.3 and −15 mmHg corresponding to −0.3 and −13% compared to baseline.

### 3.2. Effects of Exercise on Bioactive Plasma Lipids

**Figure 3:**
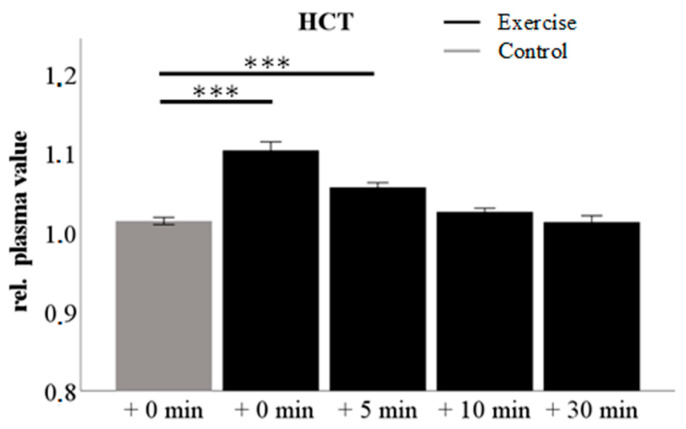
Relative changes in hematocrit compared to baseline in control (grey) and exercise intervention (black) at different time points in the early post-exercise period; *** *p* < 0.001 in comparison to the control intervention.

Blood samples were taken at the indicated time points before and after exercise intervention and plasma was collected. Corresponding control blood was withdrawn during the sedentary control intervention (see Supplementary Figure S1). The early post-exercise period is characterized by blood flow redistribution as indicated by a significant shift in the hematocrit (HCT) (Figure 3). Repeated measures ANOVA for both interventions showed a significant difference directly after exercise (compared time points: 0, 60, 120, 180 min; Mauchly-W: 0.345; *p* < 0.05; Greenhouse-Geissner corrected: test for inner-subject effects: “time point”: F = 34.4; *p* < 0.01; “time point * “intervention”: F = 38.5 *p* < 0.01; contrasts for “time point * intervention” only significant at time point “0” with F = 53.7; *p* < 0.01). Additional dependent sample t-tests revealed that the change in the hematocrit persists at +5 min after exercise (+0 min: T = −7.6; *p* < 0.001; +5 min: T = −4.9; *p* < 0.001) but diminishes at +10 min after exercise. To exclude effects on plasma lipid levels that are merely due to fluid shift, we therefore chose +10 min after exercise as the reference point (RP) to compare plasma lipid levels between exercise and sedentary control intervention.

**Figure 4:**
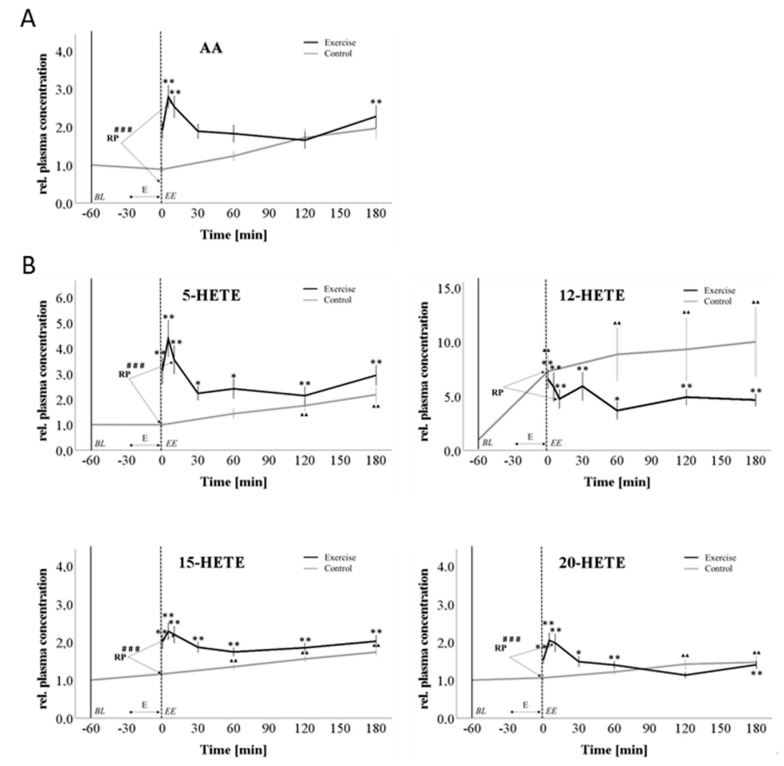

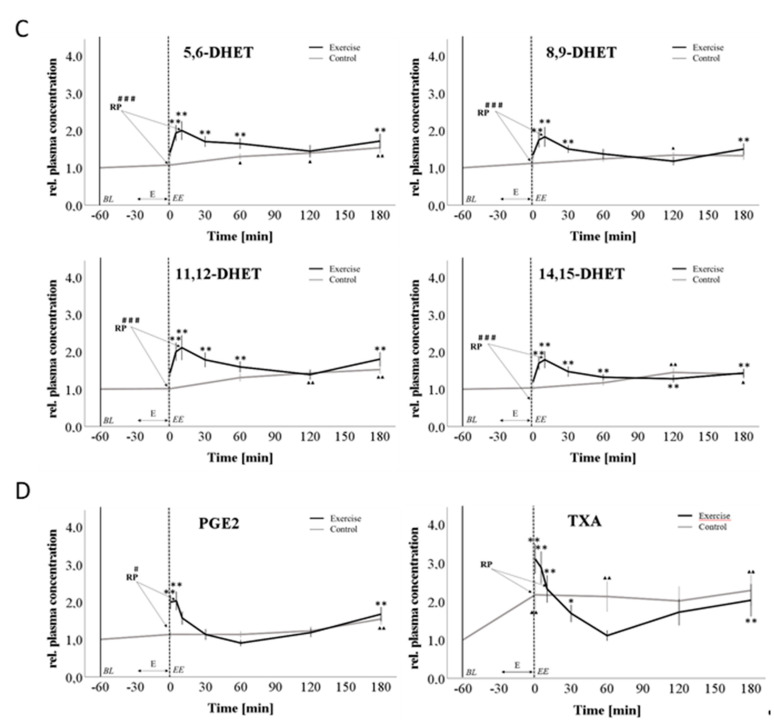
Time courses of the relative levels of arachidonic acid (**A**) and its metabolites ((**B**) HETEs, (**C**) DHETs and (**D**) prostaglandins) in plasma. Baseline (BL) values were determined at −60 min at both study days and were set as 1 for better comparison. Exercise (E) was performed between −30 min and 0 min. Since it was not possible to perform measurements during ergometer cycling, there is a gap in the exercise intervention graph during this period. black: exercise intervention grey: sedentary control intervention; values are expressed as mean fold change from baseline ± SE, ** *p* < 0.01 * *p* < 0.1 (Friedmann test with Conover-Test (post-hoc) Bonferroni-Holm corrected) significant difference vs. baseline value, # *p* < 0.05, ### *p* < 0.001 significant difference control vs exercise intervention at RP (Wilcoxon signed rank test for dependent samples), *n* = 18. Abbreviations: HETE: hydroxyeicosatetranoic acid; DHET: dihydroxyeicosatrienoic acid, PGE2: prostaglandin E2, TXB: thromboxane B, AA: arachidonic acid, EE: end of exercise; RP: reference point.

To investigate arachidonic acid and several of its metabolites as potential regulators of PEH, the plasma concentrations of these bioactive lipids were determined by LC-MS/MS analysis (Figure 4, Supplementary Tables S2 and S3).

Due to the fact that normal distribution could not be assumed for all lipids and time points, we used different approaches to statistically analyze differences in the plasma lipid levels. On the one hand, we compared lipid levels of exercise and control intervention directly at the time point +10 min, which served as the reference point (RP). Wilcoxon-signed rank test at the RP revealed significant differences between exercise and sedentary control intervention in AA, 5-HETE, 15-HETE, 20-HETE, 5.6-DHET, 8.9-DHET, 11.12-DHET, 14.15-DHET and PGE2 (Supplementary Table S4). 12-HETE and TXA showed no significant differences between the treatments.

On the other hand, we separately analyzed changes with time in the plasma lipid values during the exercise and the control interventions, in comparison to their respective baseline values, by applying the Friedmann test with Bonferroni corrections for multiple testing (Figure 4).

First of all, we determined the plasma levels of arachidonic acid (AA) and observed a strong increase after the cycling intervention with a peak at +5 min after cessation of exercise. Afterwards, the plasma lipid levels decreased rapidly until +30 min after the end of the cycling period and then remained relatively stable at an elevated concentration compared to baseline. From +120 to +180 min there was a second slight increase. During the non-exercise control intervention, we observed a slight and steady diurnal increase from baseline until the end of the experiment, which, however, was not significant (Figure 4A). The strong regulation of arachidonic acid suggested that there are also likely to be potential changes in its downstream products. Since cyclooxygenase and CYP450 products have already been associated with exercise and vasodilation, respectively, we first focused on regulation of lipoxygenase-derived HETEs (5-, 12- and 15-HETE). These HETEs showed significantly increased plasma levels directly at the end of the exercise intervention and during the early post-exercise phase. Then, levels declined quickly; however, to a level that was still elevated compared to baseline. During the non-exercise control intervention, there was a slight but constant increase in 5- and 15-HETE levels until the end of the experiment. The kinetics of 12-HETE showed different characteristics. Although the increase in plasma concentration was stronger than that found for 5- and 15- HETE, this regulation was overtaken by 12-HETE plasma levels during the non-exercise intervention at the time point +0 min. Thereafter, 12-HETE plasma levels in the non-exercise intervention further increased with high inter-individual variance, while plasma concentrations in the exercise intervention decreased to four–five-fold compared to baseline (Figure 4B). Further LC-MS/MS analyses revealed that the CYP450 epoxygenase-derived AA metabolites 5,6-; 8,9- and 11,12-DHET as well as the hydroxylase product 20-HETE also increased after the exercise intervention. 20-HETE showed similar kinetics to those of 5-HETE with a strong increase directly at the end of the cycling period. DHETs showed a significant increase starting at +5 min after exercise cessation. During the subsequent early post-exercise period, there was a rapid decrease in DHET plasma levels reaching concentrations comparable to the corresponding non-exercise levels at about +120 min. In parallel to arachidonic acid, there was a slight second increase between +120 min and +180 min. Again, the non-exercise intervention was associated with a slight but constant rise in plasma DHET (Figure 4C). PGE2 and thromboxane were detected as cyclooxygenase products of AA metabolism. During the exercise intervention, thromboxane showed a significant peak at +0 min after exercise, then decreased constantly and reached baseline values at +60 min. From +60 min towards +180 min, there was a second slight increase which reached significance at +180 min. During the course of the non-exercise control intervention, we also observed an increase in thromboxane at time point +0 min, which was weaker than in the exercise intervention and remained stable until the end of the observation period. PGE2 revealed a significant increase directly after exercise with a subsequent decrease until +60 min to below baseline (0.9-fold). Then, there was a constant increase until the end of the experiment. In the non-exercise control intervention, there was a constant time-dependent increase with a maximum at +180 min (Figure 4D).

### 3.3. Correlation Analysis

**Figure 5:**
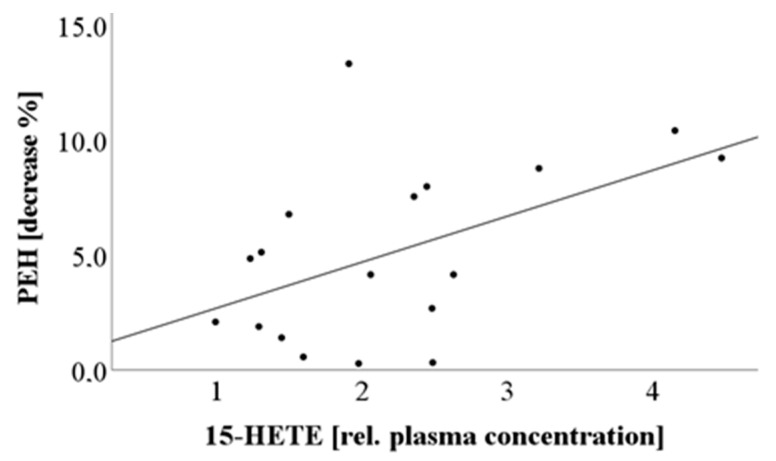
Pearson Correlation of 15-HETE plasma levels versus PEH; fold-change in 15-HETE plasma level (% decrease compared to baseline at 10 min after cessation of exercise) versus PEH.

The focus of the current pilot study was the identification of lipid mediators, in particular HETEs, which might be suitable as predictors of PEH. Therefore, a correlation analysis was performed, which revealed a significant relation between 15-HETE and the magnitude of PEH, +5 min and +10 min after cessation of exercise (Figure 5).

Additionally, a bootstrapping-calculation with 2000 samples was performed to overcome possible bias in Pearson’s correlation due to small sample size or deviations in normal distribution. The analysis revealed the following 95% BCa intervals for the time point +10 min after cessation of exercise: lower bound: 0.140; upper bound: 0.797; as this interval does not intersect with zero, these results further support a relation between PEH and 15-HETE plasma level after exercise.

Besides 15-HETE, neither AA nor its metabolites correlated significantly with PEH suggesting that there are no group effects but a specific relationship between 15-HETE and PEH (Supplementary Table S5).

To further confirm this assumption, we calculated a semi-partial correlation to evaluate possible confounding factors. The results again revealed that the effect was not due to a general AA-effect. As the relation between PEH and 15-HETE becomes even stronger after correction for AA or 5-HETE (Table 3), there may be no, or even an opposing effect of AA that needs further investigation. Only BMI may act as an interfering factor.

## 4. Discussion

Exercise is considered to be an effective therapeutic approach for the treatment of hypertension but only a fraction of patients responds to regular body activity. Post-exercise hypotension occurring acutely after exercise may represent a suitable variable to distinguish between “responders” and “non-responders,” but the underlying molecular mechanisms of this phenomenon are far from being completely elucidated. In this clinical pilot study performed with healthy normotensive volunteers, we aimed to investigate the impact of a single bout of extensive aerobic exercise on blood pressure and on a panel of different lipid mediators which might influence the vascular tone and contribute to PEH.

Our results showed that PEH is induced after endurance exercise in association with the regulation of a number of eicosanoids. Of all these regulations, a correlation with PEH could only be observed for 15-HETE, indicating that this AA metabolite might be suitable as predictor for exercise-induced blood pressure reduction.

In accordance with previous studies, induction of PEH showed high inter-individual variability. Nevertheless, the mean blood pressure reduction after 30 min was 6.1 mmHg, which is in line with decreases reported in other studies with young, healthy and normotensive individuals [39,40], but weaker than PEH observed in pre-hypertensive and hypertensive patients [41].

PEH is at least partially due to changes in local vasoactive mediators [11] such nitric oxide and prostaglandins, but the responsible mechanisms remain unclear [29,42,43]. Many bioactive lipids derived from arachidonic acid are well known for their role in vascular function and blood pressure regulation [44,45,46,47,48,49,50]. However, only a few studies have focused on these mediators in the context of exercise and fewer on their correlation with PEH. Moreover, to the best of our knowledge, thus far, there have been no studies investigating HETEs in relation to PEH. Therefore, in this study, we assessed the impact of a panel of exercise-induced AA metabolites on PEH with a focus on HETE. Using LC-MS/MS analyses with close-meshed time kinetics, we observed an increase in most plasma lipids in the early post-exercise phase. These data are consistent with previous studies which showed an increase in AA and 5-, 12-, 15- and 20-HETEs in plasma [24,27] and of 5-, 12- and 15-HETE in muscle biopsies [26] after exercise. Other reports revealed reduction of AA [23] or found no significant changes at all [25]. 5,6-DHET was found to rise after treadmill and 14,15-DHET and 14,15-EET after cycling [23,25]. Furthermore, plasma PGE2 [51] and thromboxane were increased after exercise in most approaches [24,27]. However, in comparing these differing results, it has to be considered that for the time points examined, exercise models and intensities and the sample sizes varied markedly from study to study, thus hindering the direct comparability of the results. In addition, there are reports indicating that carbohydrate intake can modify plasma lipid levels [27]. Therefore, the nutritional status of the study participants might influence data analysis but was not recorded in most studies. Interestingly, the majority of clinical studies on lipid regulation during exercise were performed without a sedentary control group and changes were normalized to baseline levels only. However, we found a rise in some lipids during the control intervention that was independent from exercise but could rather be attributed to diurnal changes. Thus, without sedentary controls, physiological regulation of lipids cannot be assessed at all and the strong increase in 12-HETE, for example, in the exercise intervention would misleadingly be judged as exercise-induced.

In our study, the strongest effects on bioactive plasma lipids were observed in the early post-exercise period. This might have been due to the rapid redistribution of blood from the muscle to the circulation. Metabolites that accumulated in the exercising muscle might then be flushed back into the circulation, thus contributing to subsequent PEH [52,53]. Another reason could be the change in shear stress in the vessel, which occurs in the direct post-exercise phase and might act as a stimulus to production or liberation of the bioactive lipids. In some cases, there was a second rise in lipids during the course of the exercise intervention, at about 2 h after cessation of exercise. This was probably due to exercise-related enzyme induction, for instance, of PLA2 or COX-2.

Correlation analyses in our study provided an indication that the 15-HETE plasma level may be a predictor of PEH in the early post-exercise period. Since AA did not significantly correlate with PEH in additional bivariate and semi-partial correlations, we suggest that the influence of 15-HETE on PEH is independent of the AA-substrate. This hypothesis was supported by the fact that no further correlation with PEH could be found for any measured lipid, indicating that a group-specific effect of arachidonic acid metabolites can be excluded. Several studies have already provided evidence for a role of 15-LOX products in blood pressure regulation [16,21,44,45]. However, the precise cardiovascular effect of 15-HETE, whether through vasoconstriction as we observed or vasodilation, is controversial since its effects depend on its concentration and the species investigated [20,21]. In addition, other 15-LOX metabolites were postulated to be inducible EDHFs [17,19,54] and contributed, for example, to acetylcholine-induced hypotension in rabbits [16,55]. These data support the role of 15-HETE as a vasodilatory effector after exercise and indicate that its effects might occur independently or in concert with other vasoactive 15-LOX products. However, it must be taken into account that this assumption relies mainly on statistical analyses of a small group of volunteers in our pilot study. Furthermore, the study participants were young and healthy and might, thus, show effects that differ from pre-hypertensive or hypertensive patients. Therefore, it will be necessary to perform further clinical studies with more male and female participants, who would ideally already show symptoms of high blood pressure.

## 5. Conclusions

Taken together, our results show that a single bout of exercise leads to a rapid accumulation of a number of bioactive lipid metabolites of the AA cascade in the plasma. The upregulation of 15-HETE correlated significantly with PEH and might be a marker of a positive prognosis for the occurrence of PEH in our trial. The increases in other metabolites failed to correlate with PEH, indicating a specific effect of 15-HETE. Since PEH is suggested to be a useful predictor for long-term efficacy of exercise training on lowering hypertension, 15-HETE might constitute a suitable biomarker for the prediction of PEH and thereby facilitate the development of individualized exercise therapy programs in hypertension therapy. As the underlying mechanism of a possible role of HETEs in PEH remains unclear, it would be relevant to further investigate inflammatory and immunological reactions in this context.

## Figures and Tables

**Table 1:** Demographic data (brackets indicate the number of male participants at the end of the study).

Demographic Data	Mean	SE
Male	5 (3)	
Female	15	
Age [years]	25.10	3.14
Height [meters]	1.72	0.07
Weight [kg]	65.38	8.88
BMI	22.12	2.43
Activity Index	55.60	26.91

**Table 2:** Mean fold-changes in systolic blood pressure in the control and the exercise intervention compared to baseline (−60 min). For a better comparison, the baseline values were set to 1; absolute values can be found in Supplementary Table S1. *** *p* < 0.001 compared to baseline of the same intervention, repeated measures ANOVA, # *p* < 0.05 comparison between control and exercise intervention (repeated measures ANOVA with pairwise comparisons of exercise and sedentary control) and Bonferroni-correction for multiple testing (*n* = 18). Abbreviations: SBP: Systolic blood pressure SE: standard error of the mean.

	SBP ± SE	
Time [Min]	Control	Exercise
**−60**	1.00 ± 0	1.00 ± 0
**0**	1.00 ± 0.01	1.02 ± 0.01
**30**	0.97± 0.01	0.95 ± 0.008 ***
**60**	1.00 ± 0.01	0.96 ± 0.01 #
**120**	1.00 ± 0.01	0.98 ± 0.01

**Table 3:** Semi-Partial Correlation of PEH with fold-changes of 15-HETE and possible confounders +10 min after cessation of exercise.

Variable 1	Variable 2	Control Variable	Pearson *r*	Significance	*n*
PEH	15-HETE	no	0.505	0.032	18
PEH	15-HETE	AA	0.744	0.001	18
PEH	15-HETE	5-HETE	0.758	0.000423	18
PEH	15-HETE	12-HETE	0.057	0.038	18
PEH	15-HETE	20-HETE	0.539	0.026	18
PEH	15-HETE	BMI	0.438	0.079	18

## Supplementary Materials

The following are available online at https://www.mdpi.com/2073-4409/9/9/2111/s1, Figure S1: Flow chart of the experimental design; Table S1: Time course of blood pressure and heart rate during exercise and control intervention (absolute measured values); Table S2: Absolute plasma lipid concentrations [ng/mL] in the control and the exercise intervention at different time points (*n* = 18); Table S3: Relative plasma concentrations of different bioactive lipids in the control intervention and the exercise intervention Table S4: Wilcoxon-signed rank test in plasma lipid levels; Table S5: Pearson Correlations of plasma lipids.
