# Supplementary material for: Exercise-Induced Changes in Bioactive Lipids Might Serve as Potential Predictors of Post-Exercise Hypotension. A Pilot Study in Healthy Volunteers
Source: Cells. 2020 Sep 16;9(9):2111. doi: 10.3390/cells9092111 (PMC7563406; doi:10.3390/cells9092111)
Supplement: Supplementary file 1 [file cells-09-02111-s001.pdf]

Supplemental material

Supplementary figure

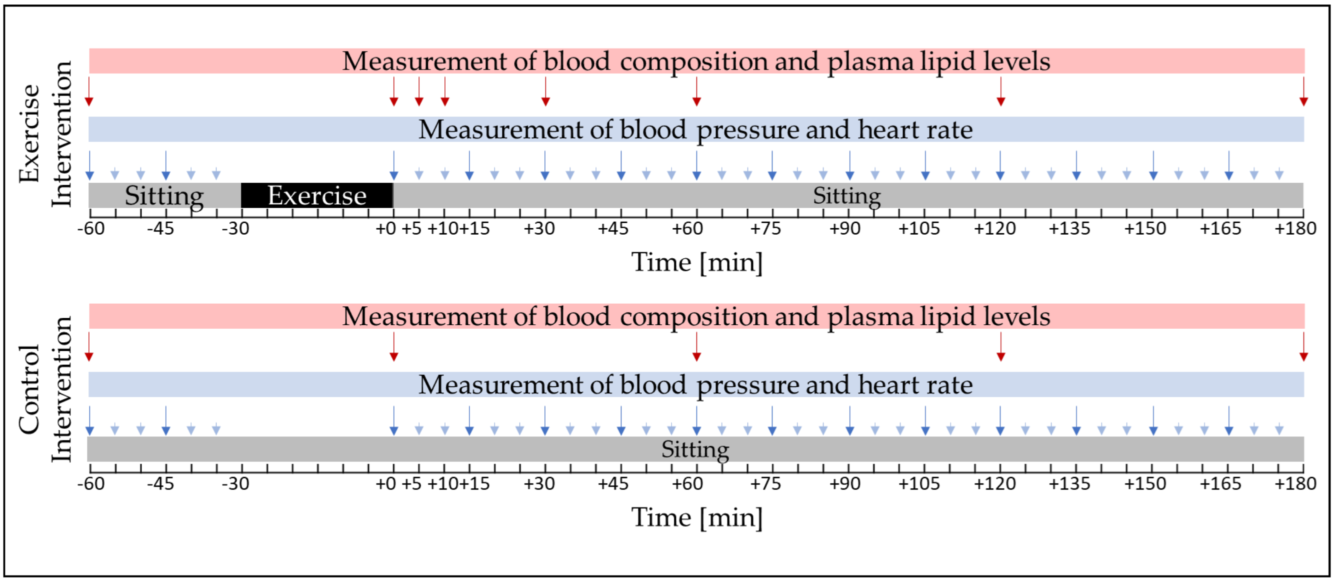

Supplementary Figure S1: Flow chart of the experimental design.

Supplementary tables

Supplementary Table S1: Time course of blood pressure and heart rate during exercise and control intervention (absolute measured values)

	SBP ± SE [mmHg]		DBP ± SE [mmHg]		HR ± SE [bpm]	
**Time Point [min]**	**Control**	**Exercise**	**Control**	**Exercise**	**Control**	**Exercise**
**-60**	119.75 ± 7.59	119.86 ± 7.82	73.86 ± 7.44	75.38 ± 6.18	74.32 ± 11.26	71.59 ± 8.35
**-45**	116.28 ± 9.15	116.72 ± 9.78	72.83 ± 8.83	73.7 ± 7.53	71.98 ± 10.95	70.57 ± 8.91
**-30**	117.25 ± 8.4		72.69 ± 6.88		72.12 ± 10.22	
**-15**	116.78 ± 7		73.8 ± 6.56		72.06 ± 9.79	
**0**	119.94 ± 9.27	122.23 ± 8.06	77.93 ± 9.36	75.89 ± 7.45	71.23 ± 12.57	91.06 ± 7.66
**15**	116.94 ± 7.76	117.88 ± 11.32	75.23 ± 6.61	75.09 ± 15.05	71.03 ± 10.11	80.98 ± 11.17
**30**	116.32 ± 6.11	113.78 ± 8.72	74.61 ± 6.63	72.02 ± 9.59	68.72 ± 10.28	77.1 ± 10.38
**45**	119.41 ± 6.75	113.48 ± 9.43	76.93 ± 6.41	72.32 ± 7.83	67.54 ± 10.61	77.65 ± 10.01
**60**	119.7 ± 7.84	115.55 ± 10.6	77.26 ± 7.41	72.85 ± 7.94	67.2 ± 9.13	75.3 ± 10.39
**75**	116.69 ± 8.43	115.52 ± 10.46	74.4 ± 6.83	71.83 ± 8.5	68.49 ± 11.01	74.73 ± 9.36
**90**	119.11 ± 8.83	115.26 ± 11.25	75.76 ± 7.13	74.12 ± 9.57	67.82 ± 12.4	72.71 ± 10.25
**105**	118.19 ± 7.86	116.29 ± 9.98	73.93 ± 6.61	73.73 ± 7.44	66.44 ± 12.26	75.32 ± 10.92
**120**	119.45 ± 7.18	117.05 ± 8.41	74.33 ± 6	75.66 ± 7.4	68.64 ± 11.79	73.61 ± 9.62
**135**	118.73 ± 7.92	117.23 ± 9.32	75 ± 8.59	74.34 ± 7.56	68.56 ± 12.6	74.01 ± 11.86
**150**	119.01 ± 10.19	117.7 ± 9.47	75.49 ± 9.42	75.23 ± 7.52	68.36 ± 10.96	71.65 ± 10.91
**165**	119.98 ± 8.58	117.64 ± 9.59	77.5 ± 7.25	74.93 ± 9.04	69.57 ± 10.82	73.63 ± 9.8

SBP: Mean systolic blood pressure [mm Hg], B: DBP: Mean diastolic blood pressure [mm Hg], C: HR mean heart rate [beats per minute, bpm]. Baseline values (BL) were determined at -60 min at both study days. Since it was not possible to perform measurements during ergometer cycling, there is a gap in the exercise intervention graph during this period. Values are expressed as means ± SE, $n = 18$.

Supplementary Table S2: Absolute plasma lipid concentrations [ng/ml] in the control and the exercise intervention at different time points (n = 18).

Analyte	5-HETE		12-HETE		15-HETE		20-HETE		5.6-DHET		8.9-DHET	
Time [min]	Control	Exercise	Control	Exercise	Control	Exercise	Control	Exercise	Control	Exercise	Control	Exercise
-60	0.09 ± 0.08	0.07 ± 0.02	0.61 ± 0.66	0.46 ± 0.46	0.17 ± 0.07	0.15 ± 0.03	0.26 ± 0.09	0.31 ± 0.16	0.04 ± 0.02	0.04 ± 0.01	0.05 ± 0.01	0.05 ± 0.01
0	0.09 ± 0.08	0.24 ± 0.21	2.28 ± 1.51	2.64 ± 2.45	0.2 ± 0.08	0.29 ± 0.11	0.27 ± 0.1	0.46 ± 0.21	0.05 ± 0.04	0.06 ± 0.02	0.05 ± 0.02	0.06 ± 0.02
5		0.32 ± 0.28		2.38 ± 2.36		0.33 ± 0.14		0.62 ± 0.38		0.08 ± 0.04		0.08 ± 0.04
10		0.25 ± 0.19		2 ± 2.2		0.31 ± 0.14		0.6 ± 0.43		0.08 ± 0.05		0.09 ± 0.06
30		0.15 ± 0.07		1.92 ± 2.06		0.27 ± 0.09		0.44 ± 0.26		0.07 ± 0.03		0.07 ± 0.02
60	0.13 ± 0.11	0.17 ± 0.1	2.01 ± 1.03	1.09 ± 0.7	0.23 ± 0.07	0.25 ± 0.07	0.32 ± 0.12	0.41 ± 0.16	0.06 ± 0.04	0.07 ± 0.03	0.06 ± 0.02	0.06 ± 0.02
120	0.15 ± 0.11	0.16 ± 0.11	2.09 ± 1.06	1.59 ± 0.86	0.26 ± 0.08	0.27 ± 0.1	0.37 ± 0.17	0.35 ± 0.18	0.07 ± 0.05	0.06 ± 0.02	0.07 ± 0.03	0.05 ± 0.02
180	0.18 ± 0.12	0.23 ± 0.15	2.31 ± 1.58	1.54 ± 0.8	0.29 ± 0.08	0.29 ± 0.11	0.38 ± 0.19	0.43 ± 0.21	0.07 ± 0.05	0.07 ± 0.03	0.06 ± 0.03	0.07 ± 0.03

Analyte	11.12-DHET		14.15-DHET		PGE2		TXS		AA	
Time [min]	Control	Exercise	Control	Exercise	Control	Exercise	Control	Exercise	Control	Exercise
-60	0.13 ± 0.09	0.13 ± 0.09	0.18 ± 0.04	0.18 ± 0.04	0.03 ± 0.02	0.03 ± 0.01	0.38 ± 0.28	0.32 ± 0.16	492.72 ± 296.87	466.76 ± 152.37
0	0.14 ± 0.11	0.19 ± 0.13	0.18 ± 0.07	0.22 ± 0.05	0.03 ± 0.02	0.05 ± 0.02	0.59 ± 0.31	0.9 ± 0.38	436.64 ± 340.92	847.41 ± 405.03
5		0.25 ± 0.16		0.31 ± 0.14		0.05 ± 0.03		0.86 ± 0.81		1237.99 ± 595.7
10		0.26 ± 0.18		0.32 ± 0.17		0.04 ± 0.02		0.66 ± 0.59		1115.33 ± 540.78
30		0.22 ± 0.17		0.26 ± 0.1		0.02 ± 0.01		0.48 ± 0.32		821.53 ± 322.52
60	0.17 ± 0.12	0.22 ± 0.19	0.21 ± 0.07	0.24 ± 0.07	0.03 ± 0.02	0.02 ± 0.01	0.52 ± 0.22	0.31 ± 0.15	592.21 ± 445.49	792.89 ± 370.51
120	0.19 ± 0.13	0.18 ± 0.11	0.25 ± 0.08	0.23 ± 0.08	0.04 ± 0.02	0.03 ± 0.01	0.52 ± 0.26	0.43 ± 0.2	770.69 ± 498.47	733.3 ± 354.14
180	0.2 ± 0.12	0.22 ± 0.15	0.24 ± 0.09	0.26 ± 0.1	0.04 ± 0.03	0.04 ± 0.02	0.59 ± 0.3	0.54 ± 0.27	855.21 ± 514.44	1034.45 ± 548.38

HETE: hydroxyeicosatetranoic acid; DHET: dihydroxyeicosatrienoic acid, PGE2: prostaglandin E2, TXB: thromboxane B, AA: arachidonic acid.

Supplementary Table S3: Relative plasma concentrations of different bioactive lipids in the control intervention and the exercise intervention (fold changes compared to baseline (-60 min), which has been set as 1 for better comparison), $^{**}p < 0.01$ $^{*}p < 0.1$ statistical significant difference compared with the baseline of the same intervention (Friedmann-Test with post-hoc Conovert-Test (Bonferroni-Holm corrected)). $\#p < 0.05$, $\#\#p < 0.01$, $\#\#\#p < 0.001$ significant difference control vs exercise intervention (two-way ANOVA with Sidak´s multiple comparison test) (n = 18).

Analyte	5-HETE		12-HETE		15-HETE		20-HETE		5.6-DHET		8.9-DHET	
**Time [min]**	**Control**	**Exercise**	**Control**	**Exercise**	**Control**	**Exercise**	**Control**	**Exercise**	**Control**	**Exercise**	**Control**	**Exercise**
**0**	0.99 ± 0.42###	3.12 ± 2.16**	7.29 ± 6.45	6.6 ± 3.67**	1.16 ± 0.29###	2.01 ± 0.77**	1.06 ± 0.34##	1.53 ± 0.39**	1.07 ± 0.36	1.41 ± 0.45	1.12 ± 0.56	1.35 ± 0.36
**5**		4.38 ± 2.98**		5.85 ± 5.58**		2.28 ± 0.95**		2.05 ± 0.84**		1.93 ± 0.89**		1.75 ± 0.83**
**10**		3.55 ± 2.34**		4.75 ± 3.74**		2.2 ± 0.94**		1.98 ± 1.01**		2 ± 1.05**		1.83 ± 1.1**
**30**		2.23 ± 1.16*		5.92 ± 5.5**		1.86 ± 0.59**		1.49 ± 0.6*		1.7 ± 0.59**		1.5 ± 0.43**
**60**	1.43 ± 0.86	2.41 ± 1.61*	8.86 ± 10.12	3.67 ± 3.39*	1.35 ± 0.28**#	1.74 ± 0.48**	1.21 ± 0.29	1.4 ± 0.42**	1.3 ± 0.35*	1.65 ± 0.58**	1.24 ± 0.33	1.37 ± 0.56
**120**	1.75 ± 0.89**	2.14 ± 1.51**	9.33 ± 12.09**	4.91 ± 3.07**	1.55 ± 0.32**	1.85 ± 0.52**	1.42 ± 0.58**	1.13 ± 0.36	1.39 ± 0.5*	1.44 ± 0.68	1.34 ± 0.35*	1.18 ± 0.45
**180**	2.18 ± 1.29**	2.94 ± 1.62**	10.02 ± 13.26**	4.65 ± 2.44**	1.73 ± 0.4**	2.02 ± 0.69**	1.47 ± 0.71**	1.41 ± 0.44**	1.53 ± 0.59**	1.71 ± 0.81**	1.32 ± 0.45	1.5 ± 0.64**

Analyte	11.12-DHET		14.15-DHET		PGE2		TXB		AA	
**Time [min]**	**Control**	**Exercise**	**Control**	**Exercise**	**Control**	**Exercise**	**Control**	**Exercise**	**Control**	**Exercise**
**0**	1.01 ± 0.23	1.44 ± 0.44	1.13 ± 0.31###	2 ± 0.78**	0.88 ± 0.28	1.91 ± 0.91##	1.03 ± 0.32	1.2 ± 0.2	0.88 ± 0.28	1.91 ± 0.91##
**5**		2 ± 1.01		2.03 ± 1.04**		2.8 ± 1.27**		1.7 ± 0.74**		2.8 ± 1.27**
**10**		2.11 ± 1.38**		1.57 ± 0.73		2.53 ± 1.2**		1.79 ± 0.93**		2.53 ± 1.2**

**30**		1.79 ± 0.82**		1.13 ± 0.59		1.89 ± 0.82		1.47 ± 0.57**		1.89 ± 0.82
**60**	1.31 ± 0.43	1.59 ± 0.61**	1.13 ± 0.37	0.9 ± 0.36	1.23 ± 0.57	1.82 ± 0.95	1.17 ± 0.31	1.32 ± 0.35**	1.23 ± 0.57	1.82 ± 0.95
**120**	1.43 ± 0.35**	1.39 ± 0.56	1.23 ± 0.51	1.18 ± 0.5	1.72 ± 1.02	1.65 ± 0.95	1.45 ± 0.51**	1.28 ± 0.39**	1.72 ± 1.02	1.65 ± 0.95
**180**	1.52 ± 0.46**	1.8 ± 0.78**	1.54 ± 0.63	1.67 ± 0.79**	1.96 ± 1.16	2.28 ± 1.23**	1.4 ± 0.53*	1.43 ± 0.49**	1.96 ± 1.16	2.28 ± 1.23**

Abbreviations: HETE: hydroxyeicosatetranoic acid; DHET: dihydroxyeicosatrienoic acid, PGE2: prostaglandin E2, TXB: thromboxane B, AA: arachidonic acid.

Supplementary Table S4. Wilcoxon-signed rank test in plasma lipid levels (fold-changes from baseline at RP (+10-min after cessation of exercise) in exercise versus fold changes from baseline at corresponding value in the sedentary control intervention).

Analyte	Z	Pearson r	Sig
**5-HETE**	-3,680	-0,867	0.000
**12-HETE**	-1,459	-0,344	0.154
**15-HETE**	-3,593	-0,847	0.000
**20-HETE**	-3,680	-0,867	0.000
**14,15-DHET**	-3,070	-0,724	0.001
**5,6-DHET**	-3,506	-0,826	0.000
**8.9-DHET**	-2,373	-0,559	0.016
**11.12-DHET**	-3,462	-0,816	0.000
**AA**	-3,724	-0,878	0.000
**PGE2**	-2,112	-0,498	0.034
**TXA**	-0,283	-0,067	0.799

Supplementary Table S5. Pearson Correlations of plasma lipids (fold-changes compared to baseline) with PEH at different time points (n = 18). Significance values of $*p < 0.05$ were considered a significant correlation of the respective analyte with PEH at the indicated time point.

	Analyte	Pearson r	Sig
+ 0 min	15-HETE	0.47	0.05
	12-HETE	-0.40	0.10
	8.9-DHET	-0.26	0.29
	5.6-DHET	0.24	0.34
	20-HETE	0.20	0.42
	5-HETE	0.17	0.51
	TXB	0.16	0.54
	14.15-DHET	0.11	0.66
	PGE2	0.11	0.67
	AA	-0.03	0.89
	11.12-DHET	0.03	0.90
+ 5 min	15-HETE	0.50	**0.03***
	PGE2	0.38	0.12
	TXB	0.37	0.14
	11.12- DHET	0.32	0.20
	8.9-DHET	0.31	0.20
	20-HETE	0.25	0.31
	5.6-DHET	0.24	0.34
	14.15-DHET	0.23	0.36
	5-HETE	0.14	0.58
	AA	-0.13	0.61
	12-HETE	0.00	0.99
+ 10 min	15-HETE	0.51	**0.03***
	TXB	0.37	0.13
	5.6-DHET	0.35	0.15
	11.12-DHET	0.33	0.18
	PGE2	0.33	0.19
	8.9-DHET	0.32	0.19
	14.15-DHET	0.32	0.20
	20-HETE	0.23	0.36
	AA	-0.20	0.42
	12-HETE	0.07	0.79
	5-HETE	-0.02	0.95

Abbreviations: HETE: hydroxyeicosatetranoic acid; DHET: dihydroxyeicosatrienoic acid, PGE2: prostaglandin E2, TXB: thromboxane B, AA: arachidonic acid.
